# Diversity and distribution of alpha satellite DNA in the genome of an Old World monkey: *Cercopithecus solatus*

**DOI:** 10.1186/s12864-016-3246-5

**Published:** 2016-11-14

**Authors:** Lauriane Cacheux, Loïc Ponger, Michèle Gerbault-Seureau, Florence Anne Richard, Christophe Escudé

**Affiliations:** 1 Département Régulations, Développement et Diversité Moléculaire, Structure et Instabilité des Génomes, INSERM U1154, CNRS UMR7196, Sorbonne Universités, Muséum national d’Histoire naturelle, Paris, France; 2 Département Systématique et Evolution, Institut de Systématique, Evolution, Biodiversité, UMR 7205 MNHN, CNRS, UPMC, EPHE, Sorbonne Universités, Muséum national d’Histoire naturelle, Paris, France; 3 Université Versailles St-Quentin, Montigny-le-Bretonneux, France

**Keywords:** Alpha satellite DNA, High-throughput sequencing, *Cercopithecus solatus*, Centromere genomics

## Abstract

**Background:**

Alpha satellite is the major repeated DNA element of primate centromeres. Evolution of these tandemly repeated sequences has led to the existence of numerous families of monomers exhibiting specific organizational patterns. The limited amount of information available in non-human primates is a restriction to the understanding of the evolutionary dynamics of alpha satellite DNA.

**Results:**

We carried out the targeted high-throughput sequencing of alpha satellite monomers and dimers from the *Cercopithecus solatus* genome, an Old World monkey from the Cercopithecini tribe. Computational approaches were used to infer the existence of sequence families and to study how these families are organized with respect to each other. While previous studies had suggested that alpha satellites in Old World monkeys were poorly diversified, our analysis provides evidence for the existence of at least four distinct families of sequences within the studied species and of higher order organizational patterns. Fluorescence in situ hybridization using oligonucleotide probes that are able to target each family in a specific way showed that the different families had distinct distributions on chromosomes and were not homogeneously distributed between chromosomes.

**Conclusions:**

Our new approach provides an unprecedented and comprehensive view of the diversity and organization of alpha satellites in a species outside the hominoid group. We consider these data with respect to previously known alpha satellite families and to potential mechanisms for satellite DNA evolution. Applying this approach to other species will open new perspectives regarding the integration of satellite DNA into comparative genomic and cytogenetic studies.

**Electronic supplementary material:**

The online version of this article (doi:10.1186/s12864-016-3246-5) contains supplementary material, which is available to authorized users.

## Background

Centromeres are chromosomal regions that control chromosome segregation during cell division in eukaryotes, through kinetochore assembly and microtubule attachment. In almost all eukaryotes, the DNA underlying centromeres is made of large tracts of nearly identical tandem DNA repeats, known as satellite DNA [1–3]. The remarkable variation of satellite DNAs between species has been an enigma ever since their discovery and different important roles have been ascribed to these sequences, from the imperative centromeric function in mitosis and meiosis to regulatory functions [4, 5].

Alpha satellite DNA is the most abundant satellite DNA in Primates and is found both at the site of centromere attachment and in neighboring heterochromatic regions, referred to as pericentromeric regions [6]. Alpha satellite DNA was originally isolated as a highly repetitive component of the *Chlorocebus aethiops* (also called African green monkey) genome [7]; homologous repeats were then described throughout the Primate order including apes, Old World and New World monkeys [8–10]. Alpha satellite DNA is made of tandemly repeated AT-rich monomers that are about 170 bp in length and organized in head-to-tail orientation [11, 12]. In the human genome, individual monomers share between 60 and 100% sequence identity. The highly identical composition of successive repeats represents a technical challenge that has thwarted the complete assembly of centromeric DNA so far [13, 14]. Nevertheless, over the last 30 years, the systematic cloning and sequencing of many alpha satellite DNAs, combined with fluorescence in situ hybridization (FISH) experiments, has provided a thorough knowledge of alpha satellite DNA diversity and organization patterns in the human genome [11, 15, 16] and, to a much lesser extent, in other primates [17–20].

In human, alpha satellite DNA has been shown to adopt two different organizations. In the so-called higher order repeat (HOR) organizational pattern, highly conserved repeat units (97–100% sequence identity), each made of multiple 171 bp monomers (up to more than 30), are found as an homogenized array that can extend over a multimegabase-sized region [2, 13, 21–23]. This organization is typically found as very long arrays of alpha satellites at the centromere core of all human chromosomes. In pericentromeres, a second type of organization, called monomeric and involving arrays of single alpha satellite monomers which are less well conserved (70–90% sequence identity), can coexist with HORs [3, 12]. Sequence comparisons between human alpha satellite monomers have led to the description of up to seventeen different alpha satellite families, or monomer types [19, 21, 24, 25]. Although the alpha satellite component of other primate genomes has been less intensively studied, there is some evidence for similar organizations in great apes, but additional families have been described and the composition of HORs as well as their chromosomal distribution differ when compared with human [12, 20, 26–28]. This implies that the structure and content of centromeric DNA can change in a few million years.

Although the mechanisms that gave rise to this diversity and organization are not precisely known, it is commonly accepted that the so-called concerted evolution of repetitive sequences is based on different mechanisms of non-reciprocal transfer occurring within or between chromosomes, such as unequal crossover, gene conversion, rolling circle replication and reinsertion, and transposon-mediated exchange [4, 29]. Such mechanisms enable series of amplification events, thereby creating new arrays of alpha satellites [12, 16, 30–32]. The analysis of the different alpha satellite families found in assembled pericentromeric regions from specific human chromosomes revealed an age gradient of the families along each chromosome arm, which led to propose that during the course of evolution, new arrays of alpha satellites expand at the centromere core, thereby splitting and displacing older arrays distally onto each arm [3, 6, 13, 19, 33].

Knowledge about alpha satellite DNA in species outside the hominoid group is very scarce, in particular in Old World monkeys, a clade that includes Colobinae, Papionini and Cercopithecini. The tribe Cercopithecini contains 35 species which have diversified within the last 10 million years [34, 35] and therefore represents a particularly interesting group for studying the evolution of satellite DNA. Moreover, it has been reported that alpha satellite DNA is more abundant in some Cercopithecini species (up to 20% of the genome of *Chlorocebus aethiops*) [36] than in great apes, where its contribution would reach only 3% of the genome [14]. Finally, enzymatic digestion of genomic DNA from various Old World monkey species can lead to a clear alpha satellite ladder pattern which is not observed when human or chimpanzee DNA is used, thereby pointing to different composition and organization of alpha satellite DNA in Old World monkeys [37].

In the present work, we have undertaken the targeted sequencing of the alpha satellite component of *Cercopithecus solatus* (or Sun-tailed monkey) as a representative species for the Cercopithecini [38]. Alpha satellite monomers and dimers were obtained by enzymatic digestion of genomic DNA and gel purification, then submitted to high-throughput sequencing. The obtained sequences were analyzed and classified into monomer families using computational approaches. Finally the genomic distribution of each family was studied by FISH using a collection of oligonucleotide probes that are able to distinguish different sequence variants. Our study provides evidence for the existence of two main families of monomers which differ in their chromosomal distribution, one being specifically distributed on centromeres while the other is found only at pericentromeric locations with a non-uniform distribution between chromosomes. Two other families are detected which are only found associated within a dimeric organization and are located for the greatest part on the Y chromosome and to a lesser extent on pericentromeres from other chromosomes. These data represent the most complete analysis of the diversity and distribution of alpha satellite sequences in an Old World monkey reported to date. Our experimental approach may be applied to other species, opening new perspectives regarding the integration of satellite DNA into comparative studies.

## Results

### Retrieval of alpha satellite sequences from the *Cercopithecus solatus* genome

Work conducted in the early 1980s had shown that enzymatic digestion of genomic DNA from Old World monkeys with several restriction enzymes resulted in a migration profile that was characteristic for alpha satellite DNA, i.e. with bands corresponding to one and multiple repeat units of about n x 170 bp in length [8, 39]. In silico analysis of several sequences isolated from *Chlorocebus aethiops* led us to select the XmnI restriction endonuclease as a candidate that should cleave a majority of monomers. Experimental digestion of *Cercopithecus solatus* genomic DNA with this enzyme revealed the expected banding pattern (Additional file 1: Figure S1). We therefore decided to extract DNA from two bands corresponding to monomers and dimers of alpha satellites from an agarose gel and implemented high throughput sequencing on an Ion Torrent sequencing platform providing reads up to 400 nucleotide in length (see Methods).

204,990 and 353,683 raw sequences were obtained for the monomer and dimer samples, respectively. Four in silico filters were applied successively to both datasets: a quality filter keeping sequences with a Phred quality score superior to 25; an extremity filter keeping sequences with the XmnI restriction site at both ends; a length filter keeping sequences within the range 162–182 bp for monomers and 324–364 bp for dimers, and an alpha satellite filter keeping sequences similar to an alpha satellite reference sequence (see Methods). The number of sequences that remained after each filter is reported on Additional file 2: Table S1. A total of 100,713 sequences fitting with all the criteria was obtained from the monomer sample and represents what we call from now on the monomer dataset. For the dimer sample, only 3,568 were obtained, they represent the dimer dataset. The drastic reduction observed within the dimer dataset was mostly the consequence of the length filter and may reflect an intrinsic limitation of the sequencing technology, unable to obtain long reads when template sequences are made of two successive highly identical sequences. These sequences were nevertheless included for further analysis as they provided an additional source of information (see below).

### Characterization of alpha satellite diversity in the monomer dataset

**Fig. 1:**
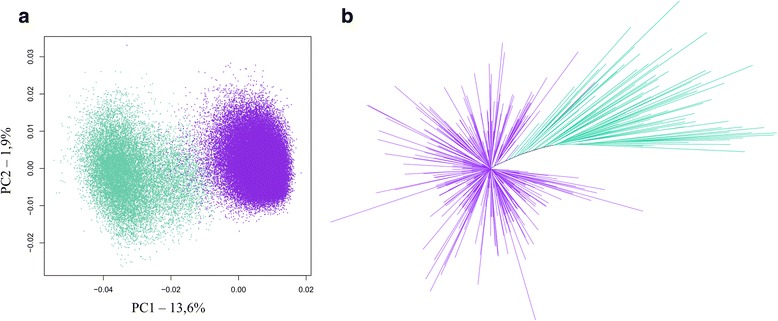
Characterization of alpha satellite DNA diversity in the monomer dataset. **a** PCA projection on principal components 1 and 2 of the normalized 5-mer frequency vectors for all sequences. Each point represents a sequence and has been colored according to its assignment to the C1 (purple) or C2 (pastel green) alpha satellite family based on hierarchical classification method. **b** Phylogenetic tree (Neighbor-joining method, K2P model) for 500 randomly selected sequences. The color code matches the one described for (**a**) and (**b**). Bootstrap values for the branches leading to C1 and C2 are not indicated as they remain below 50%

**Fig. 2:**
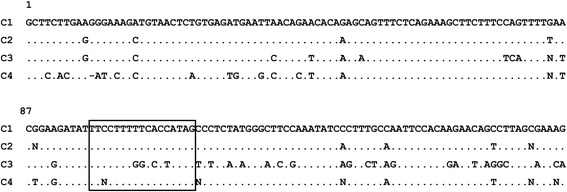
Consensus sequences of the alpha satellite families identified in the *Cercopithecus solatus* genome. The consensus sequences were determined following the alignment of 500 randomly selected sequences for the C1 and C2 families, and the alignment of the available 109 and 112 sequences for the C3 and C4 families respectively. Each position was considered unambiguous if more than 60% of monomers had the same nucleotide at this position. A point at a position replaces a nucleotide identical to the nucleotide at the homologous position in the C1 consensus. The box shows the fixation site of the pJalpha protein, which is absent from the C3 consensus

A principal component analysis (PCA) using the 5-mer nucleotide composition of DNA sequences was applied to the monomer dataset in order to compare these sequences and identify putative groups without direct alignment. Visualization of sequences into the plane formed by the two first components of the PCA revealed two main groups of alpha satellite monomers, as shown by the distribution of points on Fig. 1a. Monomers were classified into each group by using a hierarchical clustering analysis (HCA) based on a subset of sequences followed by a linear discriminant analysis (LDA) to extend the classification to all the sequences (see colors on Fig. 1a). The most important group, called C1 and shown in purple, contained 82% of the sequences and the other group, called C2 and shown in pastel green, contained the remaining 18%. To address the quality of this classification, 500 sequences were then randomly selected within the complete monomer dataset and were used to generate a phylogenetic tree where branches were colored according to the monomer classification (Fig. 1b). The disposition of sequences from the C1 and C2 groups on this tree provided a further support to our classification into two groups. Moreover, this tree showed a higher degree of divergence between C2 sequences compared to C1 sequences. Actually, the comparison of a subset of 500 randomly selected sequences within each group showed that the average sequence identity inside C1 was 95%, whereas the average sequence identity inside C2 was only 85%. The consensus sequences of C1 and C2 were 172 bp in length, and differed from each other by a total of 9 positions (Fig. 2). Finally, monomers were searched for the presence of CENP-B and pJalpha boxes [40]. A pJalpha box was present in the consensus of C1 and C2 and was found in 95% of C1 sequences and 85% of C2 sequences, whereas a CENP-B box was only found in 0.05% and 0.04% of these sequences, respectively.

In order to further characterize the sequence diversity within the monomer dataset, we searched for the presence of identical sequences and noticed that while the sequences within the C2 group were all unique, numerous identical sequences could be found within the C1 group. A total of 4,850 sequences were repeated at least twice, representing a total of 20,248 reads in our dataset. Among those sequences, 20 were repeated more than 40 times and one 2,678 times. We decided to investigate further the 20 most abundant sequences. The most abundant sequence was exactly the consensus sequence of the C1 group, while all the others corresponded to this sequence with single nucleotide variations and/or deletions, as indicated on Table 1. The absence of repeated sequences in the C2 group let us hypothesize, by contrast, that the different repeats observed in the C1 group may directly reflect the presence of strictly identical sequences in the *Cercopithecus solatus* genome. As Ion Torrent sequencing has been reported to give rise to sequencing errors, we decided to search if the identical sequences were obtained from reads collected in both orientations. We found that five out of the 20 sequences were associated with a strong bias for read orientation (Table 1). Within these five sequences, the three more abundant (2, 3, 8) represented deletions within a homopolymer tract, while the two others (15, 20) corresponded to the combination of the two most abundant deletions (found in 2 and 3) with the most abundant single nucleotide variation (found in 4). Deletions within homopolymer tracts have already been shown to be inherent to the Ion torrent Technology [41] and the orientation bias we observed let us conclude that sequences displaying these deletions were non-relevant artifacts. On the contrary, all other sequences observed in high copy number, which were all obtained in both sequencing orientations, would correspond to sequence variants that are present with a high abundance in the *Cercopithecus solatus* genome.

**Table 1:** Analysis of alpha satellite sequences found in high copy number in the monomer dataset

Id	Sequence	Number	Forward (%)
1	Consensus	2678	46
2	C114Del	486	1*
3	T101Del	357	99*
4	T39G	242	46
5	A40C	101	56
6	T121A	92	41
7	T74G	78	47
8	T80Del	78	100*
9	G84C	78	41
10	C42G	76	53
11	G1A	74	43
12	A110G	65	48
13	A112T	61	38
14	T19C	59	37
15	T39G-C114Del	57	0*
16	A151C	56	52
17	G79C	55	53
18	C89T	53	49
19	G1T	46	63
20	T39G-T101Del	41	98*

The sequences are ordered and numbered according to the number of identical copies of the sequence in the monomer dataset. The “Sequence” column indicates how each sequence differs from the consensus sequence of the C1 family, using standard notations. The “Number” column displays the number of identical copies of the sequence in the monomer dataset. The “Forward” column displays the percentage of reads obtained in the forward orientation (i.e. the orientation of our reference sequence)

Strong biases for read orientation are shown with an asterix (*)

### Characterization of alpha satellite diversity in the dimer dataset

**Fig. 3:**
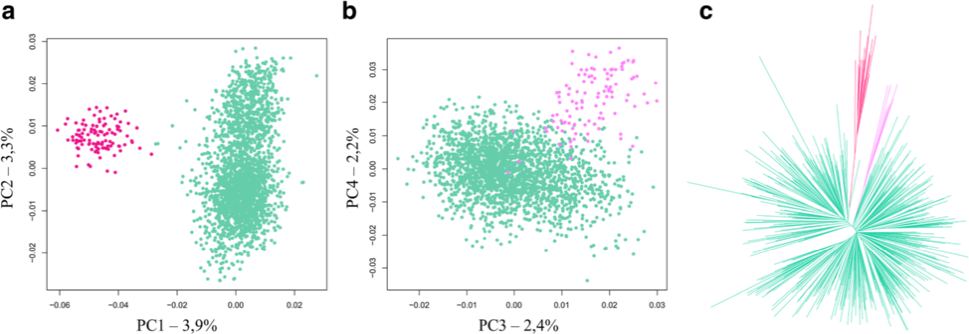
Characterization of alpha satellite diversity in the dimer dataset. PCA projection of the normalized 5-mer frequency vectors are shown for (**a**) the left monomers and (**b**) the right monomers. Each point represents a sequence and has been colored according to its assignment to the C2 (pastel green), C3 (dark pink) or C4 (light pink) alpha satellite family based on hierarchical classification method. **c** Phylogenetic tree (Neighbor-joining method, K2P model, 100 bootstraps) for 250 randomly selected left monomers and 250 randomly selected right monomers. The color code matches the one described for (**a**) and (**b**). Bootstrap values for the branches leading to C3 and C4 are 87 and 55, respectively

Among the 3,568 sequences recovered from the dimer dataset, 1,095 contained an intact XmnI restriction site approximately located in the middle, suggesting that the enzymatic digestion was not complete. The remaining 2,473 sequences, which did not possess the XmnI restriction site, were split using an alignment-based process (see Methods) and the resulting monomers were submitted to a length filter, giving a total of 2,408 associated left and right monomers. We focused first on these sequences and submitted them to the same process as described above. PCA showed the existence of two groups for both the left and right monomers, which could be discriminated using HCA (Fig. 3a and b). A comparison of the consensus sequences of each group revealed that the most abundant sequence set for both the left and right monomers belonged to the C2 group, while the two smaller sequence sets had consensus sequences that differed from each other and from the consensus of the C1 and C2 groups (Fig. 2). These two new groups of sequences, which represented 5% of the left or right monomers, will be from now on called C3 and C4 and shown in dark and light pink, respectively. We decided to build a phylogenetic tree with left and right monomers mixed together, using the described color code (Fig. 3c). This tree confirmed the existence of C3 and C4 as separate groups. Their respective average sequence identities were measured to be 86% and 83%. We also checked that left and right monomers belonging to the C2 group could not be distinguished from each other on a phylogenetic tree (Additional file 1: Figure S2), or from the C2 sequences present in the monomer dataset. The comparison of the consensus sequences showed that the C3 and C4 groups differed much more from each other and from the C1 and C2 groups than C1 and C2 differed from each other. Interestingly, C4 was the only group with a consensus length of 171 bp instead of 172 bp. A search for CENP-B and pJalpha boxes showed that most sequences within the C4 group contained a pJalpha box (75%) while the CENP-B box was absent, like observed for the C1 and C2 groups. By contrast, neither the pJalpha box nor the CENP-B box was found in the sequences from the C3 group (Fig. 2).

The dimer dataset was also used to infer information regarding how monomers belonging to different groups associated with each other. All left and right monomers were assigned to one of the C1 to C4 groups (see Methods). Additional file 2: Table S2 reports the results of these assignments as well as associations between left and right monomers, distinguishing dimers that contained the XmnI site (X dataset) and those where the XmnI site was absent (noX dataset). We noticed that sequences from the C1 group were absent from the noX dataset and were poorly represented in the X dataset. This result may appear unexpected as 82% of the sequences from the monomer dataset belonged to the C1 group. Two hypotheses may explain this observation: the high sequence identity within the C1 group may reduce both the likelihood of the inactivation of the XmnI digestion site through mutations and the sequencing efficiency of dimers (see above). A statistical analysis of the X dataset showed that left monomers from the C1 and C2 groups were preferentially associated to right monomers from the same group (Additional file 2: Table S2), which suggests that sequences from the C1 and the C2 groups are tandemly repeated in the *Cercopithecus solatus* genome. C2-C2 associations were also found to predominate within the noX dataset. Interestingly, left monomers from the C3 group were preferentially associated to right monomers from the C4 group, suggesting the existence of a higher order organization with repeats containing at least two monomers belonging to different groups.

### Genomic distribution of alpha satellite families on *Cercopithecus solatus* chromosomes

We were next interested in studying the genomic distribution of the four groups of sequences identified above. Short oligonucleotide probes have been shown to be more efficient at distinguishing alpha satellite sequences that differ by very few nucleotides compared with classical probes obtained by random priming or nick translation [42, 43]. We chose to use synthetic 18-mer oligonucleotides carrying locked nucleic acid (LNA) modifications at one out of two positions and capable of forming at least 7 GC base pairs, as previous work had demonstrated their interest for the detection of alpha satellite sequences [44]. An in silico probe selection process was implemented in order to identify among the most common 18-mer sequences within a group (found in more than 20% of the monomers) those that were specific for this group (found in less than 3% of the monomers of other groups). As we expected that oligonucleotide probes may still hybridize in the presence of one mismatch, we calculated the expected binding frequencies when one mismatch was present and applied the same selection criteria once again. Additional file 1: Figure S3 reports the sequences that best fitted with our requirements, albeit not completely. Due to the high sequence similarity between sequences within the C1 and C2 groups, probes had to distinguish sequences that differ mainly by only two nucleotides or even a single one (Additional file 1: Figure S3). The two sets of probes selected to target the C1 and C2 groups were therefore designed so that they would compete with each other if used simultaneously. The detection systems (fluorophores or haptens) were chosen in order to allow various combinations of probes to be tested together.

**Fig. 4:**
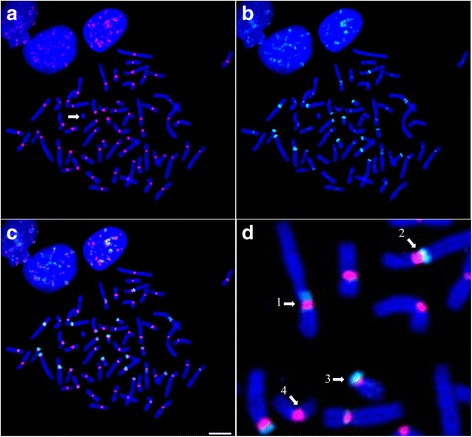
FISH analysis of the C1 and C2 alpha satellite families characterized in the monomer dataset. Probes C1b and C2b are hybridized simultaneously to *Cercopithecus solatus* chromosomes, which are colored in blue. **a** Hybridization of probe C1b is shown in red. The arrow points to a single unlabeled chromosome. **b** Hybridization of probe C2b is shown in green. **c** Combined signals from (**a**) and (**b**). (**d**) Focus on image (**c**) showing in details the different types of distribution of the C2b signals relatively to C1b. 1: C2b labels both pericentromeric regions, 2: C2b labels one pericentromeric region toward the long arm, 3: C2b labels one pericentromeric region toward the short arm of an acrocentric chromosome, 4: no C2b signal can be observed on this chromosome. Scale bar = 10 μm

A first series of FISH experiments on *Cercopithecus solatus* metaphase spreads was performed using probes C1a and C2a or C1b and C2b simultaneously. Probes targeting the C1 group produced intense signals at the centromere (primary constriction) of all chromosomes except a single one (Fig. 4a and c), while probes targeting the C2 group provided signals that are located in the pericentromeric regions (around the primary constriction) of several chromosomes pairs with different labeling patterns (Fig. 4b and c and Additional file 1: Figure S4a). Some chromosomes were extensively labeled by C2 probes on both sides of the centromere, others seem to be labeled at only one side, and others seemed to display no signal (see arrows on Fig. 4d). Stronger signals were observed on the acrocentric chromosome short arms (Additional file 1: Figure S4a). When these probes were used alone, each C1a or C2a probe produced a labeling pattern similar to what was observed in the presence of the other. On the contrary, each C1b or C2b probe used alone labeled regions that are larger than in the absence of the other. These experiments suggest that our probes may hybridize to sequences that differ by a single nucleotide (i.e. C1b binds to sequences from the C2 group and C2b binds to sequences from the C1 group) but that this binding is inhibited in the presence of an adequate competitor probe. In addition, when target sequences differ by at least two nucleotides, a specific detection is achieved in the absence of competitor.

Additional experiments showed that in presence of competitors, the signal produced by C1a overlapped with the signal produced by C1b and the signal produced by C2a almost perfectly overlapped with the one produced by C2b (Additional file 1: Figure S5). This observation supports the idea that the labeling patterns observed with the chosen oligonucleotide probes reflect the distribution of the sequence groups identified by sequence analysis. Moreover, the absence of overlap between signals provided by probes targeting sequences from the C1 and C2 groups suggests that monomers within each group are clustered together and do not mix with each other. Combined with the arguments described above that are in favor of a tandem organization of monomers for both the C1 and C2 groups, these features support the fact that the C1 and C2 groups of sequences represent distinct families of alpha satellite DNA that display a monomeric organization in the genome of *Cercopithecus solatus*.

**Fig. 5:**
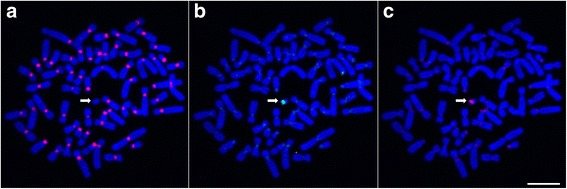
FISH analysis of the C3 and C4 alpha satellite families characterized in the dimer dataset. Probes C1a, C3a and C4a are hybridized simultaneously to *Cercopithecus solatus* chromosomes, which are colored in blue. **a** Hybridization of probe C1a is shown in red. A single chromosome (shown with an arrow) is not labeled. **b** Hybridization of probe C3a is shown in green. **c** Hybridization of probe C4a is shown in red. The pericentromeric regions of several chromosomes are sparingly labeled by C3a and C4a. Scale bar = 10 μm

Further experiments were performed with probes targeting the C3 and C4 groups. All C3 and C4 probes provided identical labeling patterns, with a strong signal located on a single chromosome, as well as very weak pericentromeric signals on some other chromosomes (Fig. 5 and Additional file 1: Figure S5). The chromosome labeled by the C3 and C4 probes, which is in fact the chromosome that was not labeled by the probes targeting C1, was also identified to be the Y chromosome by cytogenetic experiments (Additional file 1: Figure S4b). The colocalization of probes targeting the C3 and C4 groups and the absence of overlap with probes targeting the C1 or C2 group is consistent with the sequence analysis described above. These results taken together suggest thus that sequences belonging to the C3 and C4 groups represent additional families of alpha satellite DNA that display a higher order organization within the genome of *Cercopithecus solatus*. As a further control of the consistency between the results from FISH experiments and sequence analysis, we showed that a 13-mer LNA probe that was designed to target the four C1 to C4 groups of sequences (called Cx) provided signals that overlapped with the combined signals of probes targeting each group, i.e. was able to label all chromosomes within the centromeric and pericentromeric regions (Additional file 1: Figure S6).

**Fig. 6:**
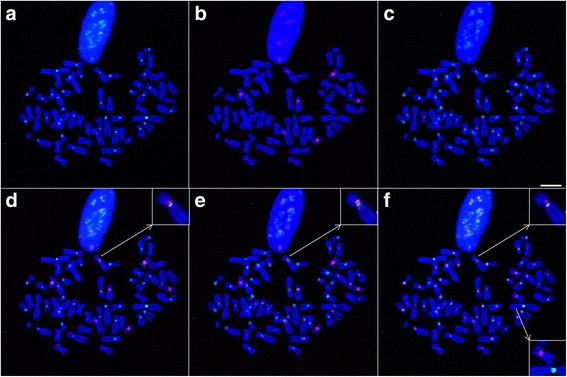
FISH analysis of the chromosomal distribution of three sequences found in high copy number. Probes T39G, A40C and C42G and the competitor oligonucleotide TACco (complementary to the C1 consensus) are hybridized simultaneously to *Cercopithecus solatus* chromosomes, which are colored in blue. **a** Hybridization of probe T39G is shown in green. **b** Hybridization of probe A40C is shown in red. Eight chromosomes are labeled. **c** Hybridization of probe C42G is shown in green. **d** Combined signals from (**a**) and (**b**). **e** Combined signals from (**b**) and (**c**). **f** Combined signals from (**a**) and (**c**) with probe T39G shown in green and probe C42G shown in red. Upper inset in (**d**), (**e**) and (**f**) shows one chromosome where signals from the 3 probes do not overlap. Lower inset in (**f**) shows two chromosomes, one being labeled by probe T39G and the other by probe A40C. Scale bar = 10 μm

We were also interested in studying the chromosomal distribution of some of the repeated sequences found in high copy number in the monomer dataset. The results of our previously described FISH experiments suggest that the specific detection of single nucleotide variations may be difficult to achieve using individual probes but that using several probes in competition may provide the possibility to achieve the required level of specificity. Therefore, we designed new oligonucleotide probes targeting a common region, aiming at distinguishing three different highly repeated sequences with single nucleotide variations (Fig. 6, see Methods). When all probes were used in combination, probes targeting sequence 4 (T39G variation) and sequence 10 (C42G variation) seemed to label all chromosomes, albeit with non-overlapping patterns (see for example insets in Fig. 6f), while probe targeting sequence 5 (A40C variation) was clearly shown to produce a signal on only 8 chromosomes.

### Comparison of *Cercopithecus solatus* alpha satellite families with known primate families

**Fig. 7:**
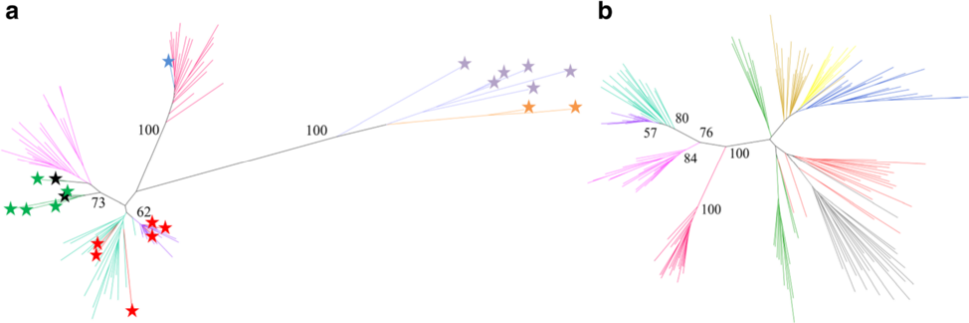
Phylogenetic relationships between *Cercopithecus solatus* alpha satellite families and other previously identified primate families. **a** Phylogenetic tree (Neighbor-joining method, K2P model, 100 bootstraps) for 20 randomly selected sequences within each C1 to C4 family and 22 monomers (labeled with stars) used in [17] to propose the S1-S5 families in Old and New World monkeys (see Methods): one S1 monomer from baboon (*blue*), two S1 monomers from macaque (*black*), five S2 monomers from macaque (*green*), six S1 monomers from *Chlorocebus aethiops* (*red*), six S4 monomers (*mauve*) and two S5 monomers (*orange*) from New World monkeys. C1 is shown in purple, C2 in pastel green, C3 in dark pink and C4 in light pink. Bootstrap values are given for principal branches when superior to 50. **b** Phylogenetic tree (Neighbor-joining method, K2P model, 100 bootstraps) for 20 randomly selected sequences within each C1 to C4 family and within pericentromeric human families: R1-2 (*blue*), M1 (*yellow*), V1 (*gold*), H1-2 (*green*), H3 (*red*) and H4 (*grey*). C1 is shown in purple, C2 in pastel green, C3 in dark pink and C4 in light pink. Bootstrap values are given for *Cercopithecus solatus* principal branches

The sequence families defined above may provide information regarding the evolutionary history of alpha satellite DNA in Primates. We were therefore interested in investigating phylogenetic relationships between these families and alpha satellite sequences that were previously described for other primate species. Interestingly, the first alpha satellite consensus sequence ever described, which was obtained for the cercopithecini *Chlorocebus aethiops* [45], was exactly the same as the consensus sequence of our C1 family, which is also the most abundant repeated sequence in our dataset. This identity suggests the conservation of the C1 family between Cercopithecini species. Although very few sequences were available, a tentative classification was previously proposed for alpha satellite DNA present in Old and New World monkeys, involving five families termed S1 to S5 [17]. We built a phylogenetic tree containing 50 sequences randomly selected within each of our C1 to C4 families and several sequences representative for S1, S2, S4 and S5 (Fig. 7a, see Methods). The S1 sequences obtained from *Chlorocebus aethiops* were intermingled in this tree with our C1 and C2 sequences. Other sequences classified in S1 but obtained from other species were dispersed in other parts of the graph, suggesting that the proposed S1 family was not relevant. There was also no clear proximity of each one of the C1 to C4 family with sequences belonging to the so-called S2, S4 or S5 family. The phylogenetic tree showed on the contrary that sequences from macaque (identified as S1 or S2) may form a sister group of the C4 family whereas the only available baboon sequence (identified as S1) was close to the C3 family. None of the sequences from macaque or baboon resembled those from our C1 or C2 family. All these results suggest that, contrary to S4 and S5, S1 and S2 do not correspond to alpha satellite families. We also built a phylogenetic tree involving our C1-C4 families and seven families (termed M1, R1-2, V1, and H1 to H4) that were previously identified in human pericentromeric regions, some of them being reported as similar to sequences found in other primates [19] (Fig. 7b). The tree suggests that alpha satellite families found in *Cercopithecus solatus* have an evolutionary history that is largely independent from that of the alpha satellite families found in human pericentromeric regions.

## Discussion

Despite the recent generalization of high-throughput sequencing, application of these new technologies to the study of repeated DNA remains scarce [46, 47]. Here, we present an original experimental and computational framework for studying repeated DNA. We have focused on a single Cercopithecini species where the diversity and organization of alpha satellite DNA are described in details. Our approach relies on sequencing of gel purified alpha satellite monomers and dimers obtained by restriction enzyme digestion of genomic DNA, followed by sequence analysis and FISH experiments with carefully designed probes.

We detected four alpha satellite families, called C1 to C4, in the *Cercopithecus solatus* genome. Additional families may have been missed by our approach, for example because they would not contain restriction sites for XmnI. Although some technical issue had drastically reduced the number of available amount of sequences containing two monomers, the dimer dataset provided information about the structural organization of each family, showing that the C1 and C2 families adopt a monomeric organization, while C3 and C4 would associate into HORs. Our data do not allow concluding if the C3-C4 dimers are tandemly repeated or represent only a part of a longer HOR involving other monomers, but suggest that such structures, which have also been observed in New World monkey genomes [48], may be widespread in Primates. It had previously been reported, using a limited number of sequences, that alpha satellite sequences in Old World monkeys contained a pJalpha binding site and no CENP-B binding site [22, 49, 50]. Our data provide further support to this observation which holds true for three of the four newly identified families. The absence of any of these two binding sites in the C3 family represents an oddity but one should notice that as sequences from the C3 family are associated with sequences from the C4 family into a HOR organization, the pJalpha binding site remains present in the repeated motif. We detected several sequences in our dataset that were repeated identically a high number of times (up to several thousands). As our protocol does not contain any PCR amplification before capture of individual sequences on beads, the abundance of these sequences may reflect their natural abundance within the *Cercopithecus solatus* genome, provided one is able to identify potential artifacts resulting from sequencing errors among those sequences.

The high similarity between *Cercopithecus solatus* alpha satellite families, especially C1 and C2, the consensus of which differ at only a few nucleotide positions, required the implementation of a highly specific FISH detection to infer their chromosomal distribution. Our results emphasize the interest of short LNA-modified oligonucleotide probes that are here shown to be able to distinguish sequences that differ by only two nucleotides. It is even possible to distinguish a single nucleotide variation between two sequences by using two probes targeting each sequence variant simultaneously. In all our experiments, we cannot exclude the possibility that probes also hybridize to sequences that are not perfectly complementary, nor that some signals do not come from sequences that are present in the *Cercopithecus solatus* genome but not found in our datasets. Nevertheless, the absence of cross labeling between the probes targeting different families and the consistency of hybridization results with predictions inferred from sequence analysis support our probe design strategy and stands for the accuracy and the exhaustiveness of our description of the alpha satellite component of *Cercopithecus solatus*.

Our FISH experiments showed that the C1 family, which is the most conserved (95% mean sequence identity), displays a centromeric localization while the more divergent C2 family (85% mean sequence identity) displays a pericentromeric localization. According to the age-gradient based model for centromere evolution [3, 33], we may speculate that this pattern results from a peculiar evolutionary history where the C2 family, an old family of sequences, had occupied a centromeric position in an ancestor of *Cercopithecus solatus.* This family would then have been displaced towards pericentromeric regions following the amplification of more recent sequences from the C1 family at the centromere. Unequal crossing over at nearly identical repeats is thought indeed to lead to the homogenization of the core centromere, while mutations would only affect repeats outside of the core centromere [3, 19, 51–53]. An alternative but non-exclusive hypothesis would attribute distinct functional roles to both families, for example centromere function to C1 and cohesion of sister chromatids to C2, as it has been proposed for mouse minor and major satellite sequences, respectively [54]. Interestingly, acrocentric chromosome short arms display a very large amount of C2 sequences as revealed by intense FISH signals. This observation provides support to a previous hypothesis according to which acrocentric chromosomes may physically interact and exchange genetic material [55, 56]. The fact that the C3-C4 dimers are found on the Y chromosome and are almost absent from other chromosomes may be explained by the fact that the Y chromosome is excluded from recombination events with non-homologous chromosomes, as was observed in mice [57]. Finally, the observation of the distribution of one of the highly repeated sequence variants on only 8 chromosomes supports the existence of local alpha satellite homogenization events in the *Cercopithecus solatus* genome.

Previous studies had considered alpha satellite DNA in Cercopithecini as poorly diversified [17]. Our results show that at least four alpha satellite families can be present in a single species, with complex chromosomal distribution and organizational patterns. Comparative studies including repetitive DNAs from different species have already been shown to provide new insights into genome and species evolution [58]. Our approach will permit not only to investigate the taxonomic distribution of alpha satellite families but also to study their organizational pattern, their chromosomal distribution as well as the existence of conserved highly repeated sequence variants. Phylogenetic analysis have demonstrated that the C1 to C4 families represent newly identified entities that do not correspond to previously proposed alpha satellite families. Although the available data are in favor of an apparent conservation of both the C1 and C2 families between *Cercopithecus solatus* and *Chlorocebus aethiop*s, further studies will be required to better understand the dynamics of alpha satellite DNA in Old World monkeys and in other primates.

## Conclusions

In summary, we have presented here a generally applicable strategy that provides, for a single species, a comprehensive description of alpha satellite sequence diversity and organization. Our approach, which is easy to implement and cost-effective, provides an opportunity to characterize satellite DNA in all species where a characteristic enzymatic ladder pattern can be obtained. Comparing different individuals and different species will provide new insights into the dynamics at which new satellite families or new highly repeated sequence variants appear during the course of evolution and transfer between chromosomes. The better description of the structure of heterochromatic regions also provides potential for enhancing the epigenetic characterization of these regions as well as understanding the regulatory functions of heterochromatin.

## Methods

### DNA collection and metaphase preparations

Fibroblast samples of *Cercopithecus solatus* (ID: 2012–028, male sample, ethic permission n° FR1207510445-I) from the Collection of cryopreserved living tissues and cells of vertebrates (RBCell collection, Muséum national d’Histoire naturelle, Paris) were used for DNA extraction and metaphase preparations. DNA was extracted using the Omega Biotek Tissue DNA Kit (Doraville, USA). Cell cultures and metaphase preparations were achieved according to [59].

### Alpha satellite DNA isolation and sequencing

The Serial Cloner software (Serial Basics, serialbasics.free.fr) was used to perform in silico digestions of the Cercopithecini alpha satellite sequences registered as such in Genbank (Accession numbers: AM235889, AM235890, AM237210, AM237214, AM237213, AM237212, X04339, V00145, M26844 and AM237211), which contained both monomers and dimers. The restriction site of the XmnI restriction enzyme (GAANNNNTTC) was observed once in a great proportion of monomers and twice in almost all dimers. XmnI was then used to digest *Cercopithecus solatus* DNA in vitro. 10 μg of *Cercopithecus solatus* genomic DNA were digested for 4 h 30 min at 37°C with 60 units of XmnI activity (New England Biolabs) in a total volume of 34 μL. The enzyme was inactivated for 20 min at 65°C. The sample was loaded on a 1% agarose gel after addition of 6.8 μL loading buffer (50% glycerol) and electrophoresis was performed in 0.5X Tris-borate-EDTA buffer, at room temperature for 2 h 45 min at 100 V. The gel was briefly stained with ethidium bromide and then imaged by UV transillumination. Bands corresponding to alpha satellite monomers (~170 bp) and dimers (~340 bp) were cut and DNA was extracted from the gel with the Omega Biotek Gel extraction kit and resuspended in 100 μl of elution buffer. About 220 ng and 110 ng were obtained for the 170 bp and 340 bp samples, respectively.

Sequencing was performed on a PGM sequencing platform (Ion Torrent technology) using the 400 bp sequencing kit. Two libraries were generated using 50 ng of both blunt digest pools and the Ion Plus Fragment Library Kit (4471252, Life Technologies) and tagged with Ion Xpress barcode adapters (4471250, Life Technologies). After purification (1.8X) with Ampure XP Beads (A63880, Agencourt Bioscience, Beverly, USA), the libraries were quantitated using a SsoAdvanced Sybr Green qPCR assay (Biorad, Hercules, USA) based on a custom *E. coli* reference library. After a dilution of each library down to 26 pM, 0.22 fmol for the 170 bp library and 0.44 fmol for the 340 bp library were pooled as templates for the clonal amplification on Ion Sphere particles during the emulsion PCR, performed on a One Touch2 emPCR robot according to the Ion PGM Template OT2 400 Kit user guide (4479878, Life Technologies). The amplification products were loaded onto an Ion 316v2 chip (4483324, Life Technologies), and subsequently sequenced according to the Ion PGM Sequencing 400 Kit user guide (4482002, Life Technologies). After standard filtration of the raw reads (polyclonal and low quality removal), the Ion Torrent sequencing yielded 204,990 sequences for the 170 bp pool and 353,683 sequences for the 340 bp pool. They were deposited in the NIH Short Read Archive (SRA accession numbers SRX1595681 and SRX1595679).

### Alpha satellite sequence filtering

All sequences with an average Phred score lower than 25, a length outside the range 162–182 bp for monomers and 324–364 bp for dimers, and sequences without the XmnI digested sites at the extremities (5′-NNTTC … GAANN-3′) were not considered for further analysis. Alpha satellite sequences were identified with a BLAST search against a reference alpha satellite sequence of *Chlorocebus aethiops* (AM23721) [60]. Using default BLAST parameters, all sequences exhibiting a hit longer than 80 bp for monomers and 160 bp for dimers were considered as alpha satellite sequences and conserved for the following analysis. All sequences were then reoriented if necessary in order to match the orientation of the reference alpha satellite sequence. The orientation information was preserved for investigations regarding reading biases.

Processing of dimeric sequences was performed as follows. When an XmnI site was present in the middle of these sequences, it was used for separating both monomers, providing the so-called left and right monomers located on the 5′ side and on the 3′ side of the sequence, respectively. Dimers that did not contain any XmnI site in the middle were aligned against a synthetic sequence formed by two consecutive copies of the reference sequence using the Needleman-Wunsch algorithm [61] to identify the monomer limits and split them into left and right monomers according to the same rule as described above. All pairs with at least one monomer outside the 162–182 bp range were discarded. Pairing information was conserved to study association between left and right monomers.

### Alpha satellite sequence characterization

Monomeric sequences were compared using their 5-mer composition in order to identify putative alpha satellite groups without direct alignment. For each set of monomers, the 5-mer frequency table was analyzed using a principal component analysis (PCA) to reduce the space complexity and enable data visualization on the first factorial planes. Sequences were classified into groups by using a hierarchical clustering method (HCA) based on the Ward criterion [62] applied to the Euclidean distances calculated from the 100 first principal components of the PCA. Because of the size of the monomer dataset, direct classification of the sequences using HCA was not possible. Instead, HCA was applied on 2,500 randomly selected sequences which were used to train a linear discriminant model. This model has been finally used to classify all the other monomers. The dimer dataset was analyzed in two different ways: 1) monomers extracted from dimers without XmnI sites were classified by using an HCA based on a PCA, 2) monomers extracted from dimers with a XmnI site have been classified by using a LDA trained to recognize the C1-C4 groups.

Because of the size of the datasets, the phylogenetic trees, the consensus sequences and the sequence distance analysis were conducted with different subsets of randomly selected sequences, using a homemade python script. The selected sequences were aligned using MUSCLE [63] and analyzed with SeaView [64]. The phylogenetic trees were built by using the Neighbor joining algorithm and the Kimura 2-parameters distance. Reliability of nodes was assessed using 100 bootstrap iterations. The relatively low bootstrap values observed in the trees can be explained by a limited number of family specific sites, i.e., the informative sites, into the alignments. Nevertheless, the same clustering of the families and the same relationship between these families have been observed with all the trees generated with different randomly selected sequences.

CENP-B and pJalpha boxes were searched with the patterns TTCGTTGGAARCGGGA and TTCCTTTTYCACCRTAG respectively [40] by using the program Fuzznuc [65] and allowing 2 mismatches. All statistical analyses were conducted with R [66]. Our R scripts and other programs are available upon request.

The S1-S5 monomers used in Fig. 7a have been isolated from the sequences described in [17]. All these monomers have been extracted by using the homologous position of the XmnI digestion site as a starting point (XmnI phase) in order to be aligned with the monomers of *Cercopithecus solatus*. Unfortunately, no full length S3 monomer was available in this phase. To obtain the Genbank accession numbers and the alignment of the used S1-S5 monomers, see [17] and Additional file 3: Text S1. Human monomers from old and ancient families (M1, R1-2, V1, H1-H4) used in Fig. 7b have been isolated from the human Xp chromosome sequence (Genbank ID NT_011630) by using the homologous position of the XmnI digestion site as a starting point. Monomers have been assigned to a family according to their location along the sequence and the annotations provided in [19] (see Additional file 4: Text S2 for alignment).

### Oligonucleotide probes

Short oligonucleotide probes (18 nucleotides in length) were designed in order to target specifically the different alpha satellite families identified in *Cercopithecus solatus*, by systematic prediction of binding frequencies based on the sequencing results. In some instances, when the 18-mer sequence did not allow forming at least 7 GC bp upon hybridization to the complementary strand, length was increased to 19. Sequences and binding frequencies are available in Additional file 1: Figure S3, which also provides details about the positions of locked nucleic acid (LNA) modifications in the probes. These positions were selected based on previous experience in order to achieve a good binding affinity and specificity [44]. When possible, we selected probes that were perfectly complementary to more than 20% of the sequences from the target group and to less than 3% of the sequences from the other groups. Additional file 1: Figure S3 also provides the expected binding frequencies if hybridization is possible despite the presence of one mismatch between the probe and its target. To target three sequences found in high copy number in the monomer dataset, we designed four LNA-modified probes (LNA are written in lower case and classic nucleotides are written in upper case): probe T39G (5′TgTtCtGtTCaTtCaTcTc3′, 5′AlexaFluor488), probe A40C (5′TgTtCtGtGAaTtCaTcTc3′, 3′Digoxygenin), probe C42G (5′TgTtCtCtTAaTtCaTcTc3′, 3′Biotin) and probe TACco (5′TgTtCtGtTAaTtCaTcTc3′) which is complementary to the C1 consensus sequence. LNA-modified probes were purchased from Eurogentec (Seraing, Belgium).

### FISH experiments

FISH were performed on metaphase chromosome preparations. Hybridization solutions were prepared by diluting the oligonucleotide probes to a final concentration of 0.1 μM in a hybridization solution consisting of 2X SSC pH 6.3, 50% deionized formamide, 1X Denhardt solution, 10% dextran sulfate, and 0.1% SDS. 20 μL of the hybridization solution were deposited on each slide and covered with a coverslip. The slides were then heated for 3 min at 70°C and hybridized for 1 h at 37°C in a Thermobrite apparatus (Leica Biosystems). Then, each slide was washed twice in 2X SSC at 63°C. Preparations were then incubated in blocking solution (4% bovine serum albumin (BSA), 1X PBS, 0.05% Tween 20) for 30 min at 37°C to reduce nonspecific binding. Then, depending on the combination of probes, the following antibodies were used for subsequent revelations: Alexa 488-conjugated streptavidin (1:200; Life Technologies, Foster City, USA), Cy5-conjugated streptavidin (1:200; Caltag Laboratories, Burlingame, USA), FITC-conjugated sheep anti-digoxigenin (1:200; Roche, Lewes, UK), and Rhodamine-conjugated sheep anti-digoxigenin (1:200; Roche). All antibodies were diluted in blocking solution containing 1X PBS, 0.05% Tween 20, and 4% BSA. Antibody incubation lasted for 30 min at 37°C. All washings were performed in 2X SSC, 0.05% Tween 20. Chromosomes were counterstained with DAPI (4′,6-diamidino-2-phenylindole) by pipetting 40 μL of a 5 μg/mL solution onto the slides, incubating for 5 min and then briefly washing in 1X PBS. Slides were mounted by adding a drop of Vectashield Antifade Mounting Medium (Vector Laboratories, Burlingame, USA) and covering with a coverslip.

### Image acquisition and analysis

Metaphases were imaged using an Axio Observer Z1 epifluorescent inverted microscope (Zeiss) coupled to an ORCA R2 cooled CDD camera (Hamamatsu). The Axio Observer Z1 was equipped with a Plan-Apochromat 63x 1.4 NA oil-immersion objective and the following filters set: 49 shift free for DAPI (G365 / FT395 / BP445/50), 38 HE shift free for FITC/Alexa488 (BP470/40 / FT495 / BP525/50), homemade sets for Rhodamine (BP546/10 / FF555 / BP 583/22) and for Cy5 (BP643/20 / FF660 / BP684/24). The light source was LED illumination (wavelengths: 365 nm, 470 nm or 625 nm) except for Rhodamine, for which a metal halide lamp HXP120 was preferred. Immersion oil of refractive index 1.518 at 23°C was used. Color-combined images were reconstructed using ImageJ [67]. At least ten metaphases were visualized for each experiment, which all confirmed the described patterns.

## Additional files

Additional file 1: Figure S1. Migration profiles of *Cercopithecus solatus* genomic DNA digested with XmnI. **Figure S2.** Phylogenetic tree for left and right alpha satellite monomers from the dimer dataset. **Figure S3.** LNA-modified probes used to target the C1 to C4 alpha satellite families on *Cercopithecus solatus* chromosomes. **Figure S4.** Distribution pattern of C2 and C3 alpha satellite families on *Cercopithecus solatus* chromosomes. **Figure S5.** Comparison of FISH signals from different probes targeting identical alpha satellite families. **Figure S6.** Comparison of the hybridization pattern of the Cx probe with those of probes targeting the C1 to C4 alpha satellite families. (DOCX 1508 kb)
Additional file 2: Table S1. Filtering steps from *Cercopithecus solatus* raw data to alpha satellite monomer and dimer datasets. **Table S2**. Alpha satellite family associations in *Cercopithecus solatus* dimer dataset. (DOCX 40 kb)
Additional file 3: Text S1. Alignment used to generate the phylogenetic tree from Fig. 7a. (TXT 28 kb)
Additional file 4: Text S2. Alignment used to generate the phylogenetic tree from Fig. 7b. (TXT 48 kb)
