# Supplementary material for: Diversity and distribution of alpha satellite DNA in the genome of an Old World monkey: Cercopithecus solatus
Source: BMC Genomics. 2016 Nov 14;17:916. doi: 10.1186/s12864-016-3246-5 (PMC5109768; doi:10.1186/s12864-016-3246-5)
Supplement: Additional file 1: Figure S1. — Migration profiles of Cercopithecus solatus genomic DNA digested with XmnI. Figure S2. Phylogenetic tree for left and right alpha satellite monomers from the dimer dataset. Figure S3. LNA-modified probes used to target the C1 to C4 alpha satellite families on Cercopithecus solatus chromosomes. Figure S4. Distribution pattern of C2 and C3 alpha satellite families on Cercopithecus solatus chromosomes. Figure S5. Comparison of FISH signals from different probes targeting identical alpha satellite families. Figure S6. Comparison of the hybridization pattern of the Cx probe with those of probes targeting the C1 to C4 alpha satellite families. (DOCX 1508 kb) [file 12864_2016_3246_MOESM1_ESM.docx]

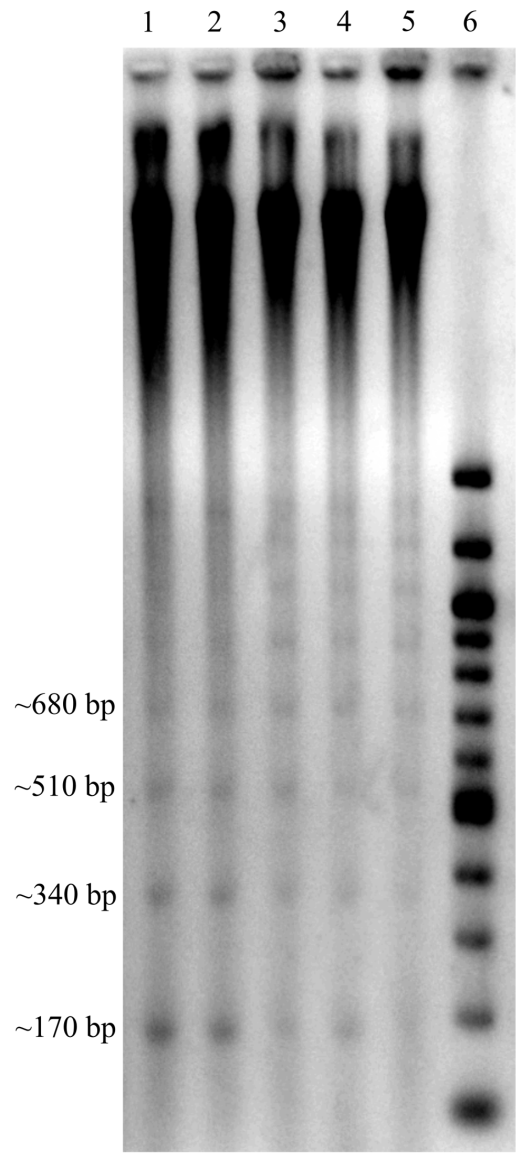


**Figure S1. Migration profiles of *C. solatus* genomic DNA digested with XmnI.** *Cercopithecus solatus* genomic DNA was digested for 1 h at 37°C with the enzyme XmnI (New England Biolabs) in a total volume of 10 µL. The enzyme was inactivated for 20 min at 65°C. The samples were loaded on a 1.5 % agarose gel after addition of 2 µL loading buffer (50% glycerol) and electrophoresis was performed in 0.5X Tris-borate-EDTA buffer, at room temperature for 2 h 45 min at 100 V. **1**: 0.5 µg of genomic DNA digested with 20 units of XmnI activity; **2**: 0.5 µg of genomic DNA digested with 10 units of XmnI activity; **3**: 0.5 µg of genomic DNA digested with 5 units of XmnI activity; **4**: 0.5 µg of genomic DNA digested with 1.5 units of XmnI activity; **5**: 0.5 µg of genomic DNA digested with 1 unit of XmnI activity; **6**: 100 bp DNA Ladder (New England Biolabs).


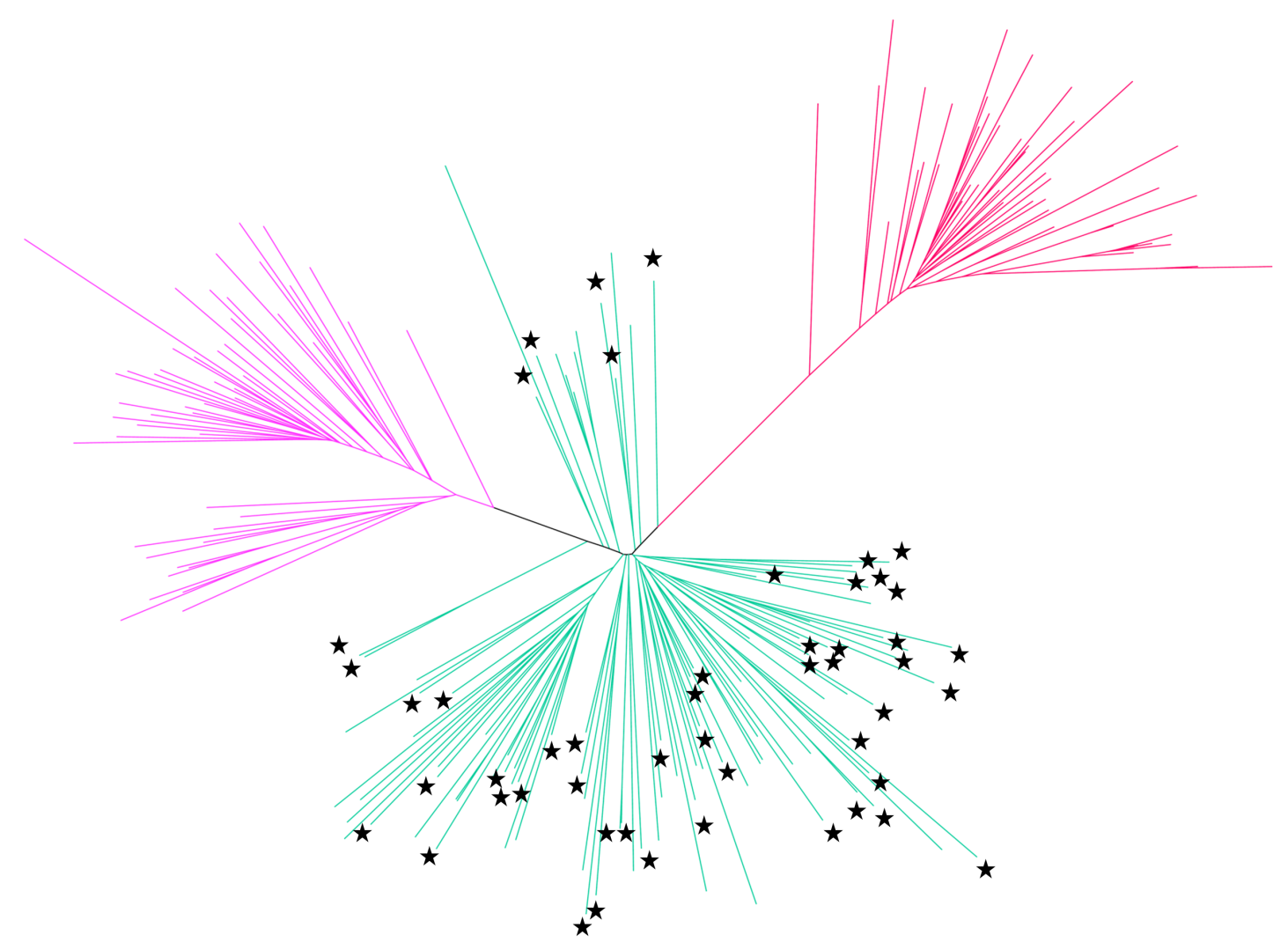


**Figure S2. Phylogenetic tree for left and right alpha satellite monomers from the dimer dataset.** The phylogenetic tree (Neighbor-joining method, K2P model) was generated following a MUSCLE alignment (Edgar 2004) between 50 randomly selected C3 (dark pink) monomers, 50 C4 (light pink) monomers, 50 C2 (pastel green) left monomers and 50 C2 (pastel green) right monomers. C2 right monomers are pointed out with a black star so we can observe that they mix well with the C2 left monomers. Only dimers without the XmnI site (noX dataset) were considered in this analysis.

Edgar RC. 2004. MUSCLE : a multiple sequence alignment method with reduced time and space complexity. BMC Bioinformatics 5(1):1.

A


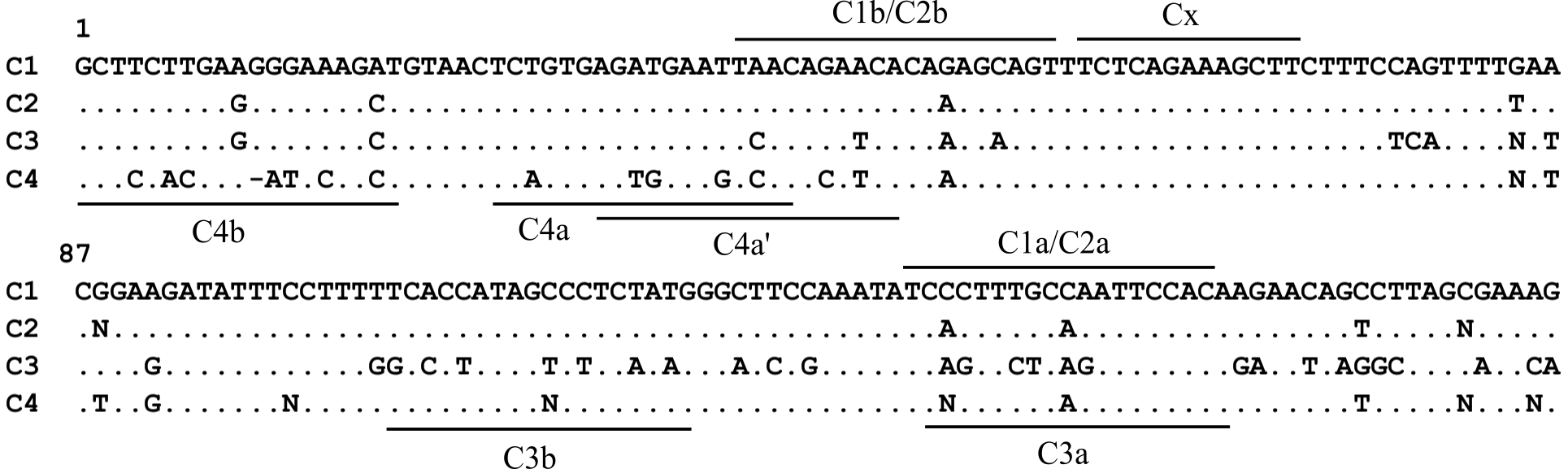


B

**Figure S3. LNA-modified probes used to target the C1 to C4 alpha satellite families on *C. solatus* chromosomes.** (A) Probe binding sites on C1, C2, C3 and C4 consensus. (B) Probe sequences and labels, associated to the percentage of targeted sequences within the C1 to C4 families. LNA are written in lower case and classic nucleotides are written in upper case. Percentage calculations have been made using all C1 and C2 sequences from the monomer dataset and all C3 and C4 sequences from the (noX) dimer dataset, when no mismatch or one mismatch was present. The sign “-“ means 0.

**
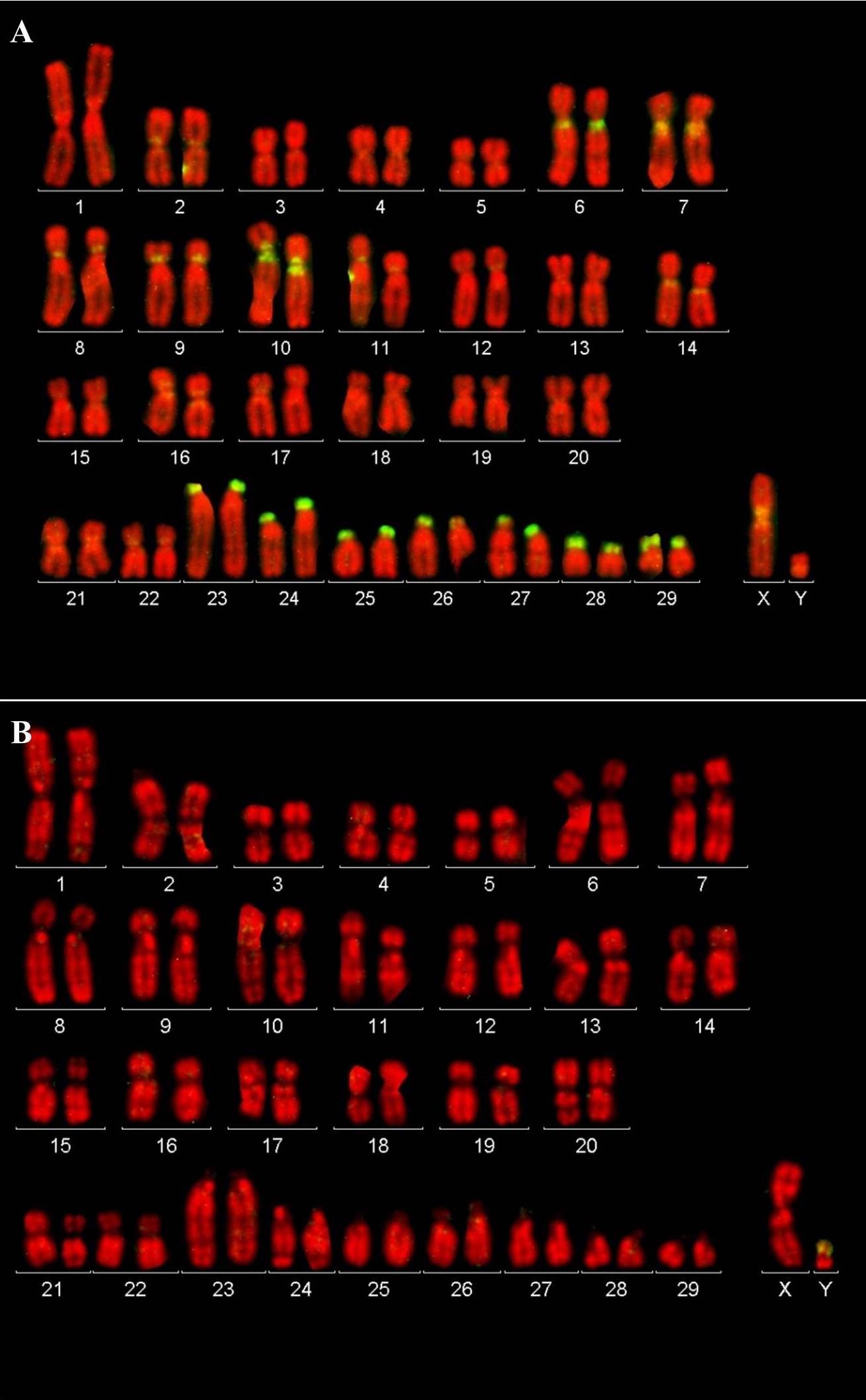
**

**Figure S4. Distribution pattern of the C2 and C3 alpha satellite families on *C. solatus* chromosomes.** Metaphase chromosomes are counterstained with propidium iodide (2µg/mL) and shown here in red, while the green colors stand for probes targeting the C2 family (C2a, shown in A) or the C3 family (C3a, shown in B). C2 signals on the long arm of chromosome 2 (right) and 11 (left) are due to chromosome superposition in the original metaphase. RBG chromosome banding techniques were employed as in Moulin et al. 2008. Observation was performed with an epifluorescent microscope (Microphot-FXA, Nikon) and images were captured using a cooled CCD camera (ProgRes MFcool, Jenoplik). The metaphases were karyotyped using the Isis 5.3 software (Metasystems, Altussheim, Germany) according to Dutrillaux et al. 1988.

Dutrillaux B, Dutrillaux AM, Lombard M, Gautier JP, Cooper R, Moysan F, Lernould JM. 1988. The karyotype of *Cercopithecus solatus* Harrison 1988, a new species belonging to *C. lhoesti*, and its phylogenetic relationships with other guenons. J. Zoo. 215(4):611-617.

Moulin S, Gerbault-Seureau M, Dutrillaux B, Richard FA. 2008. Phylogenomics of African guenons. Chromosom. Res. 16(5):783-799.


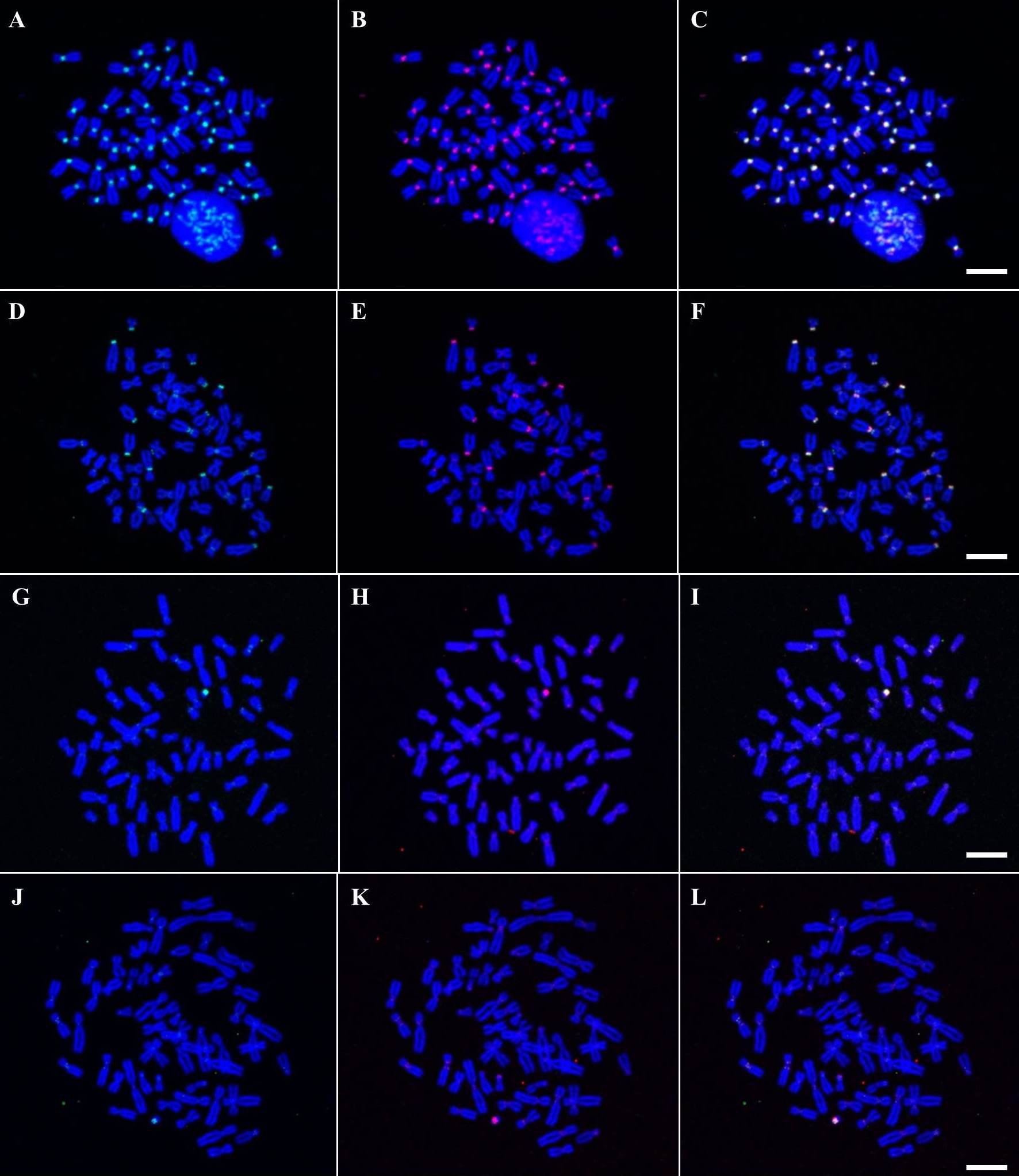


**Figure S5. Comparison of FISH signals from different probes targeting identical alpha satellite families.** Probe mixtures were hybridized as indicated to *C. solatus* metaphase chromosomes, which are colored in blue. Probe signal is shown in green (A, D, G and J) or red (B, E, H and K). Images shown in C, F, I and L are superpositions of the two previous images on the same lane. Probe mixtures are the following ones: (A,B,C) C1a (green), C1b (red) and C2b (not shown), (D,E,F) C2a (green), C2b (red) and C1b (not shown), (G, H, I) C3a (green) and C3b (red), (K,L,M) C4a’ (green) and C4b (red). Scale bar = 10 µm.


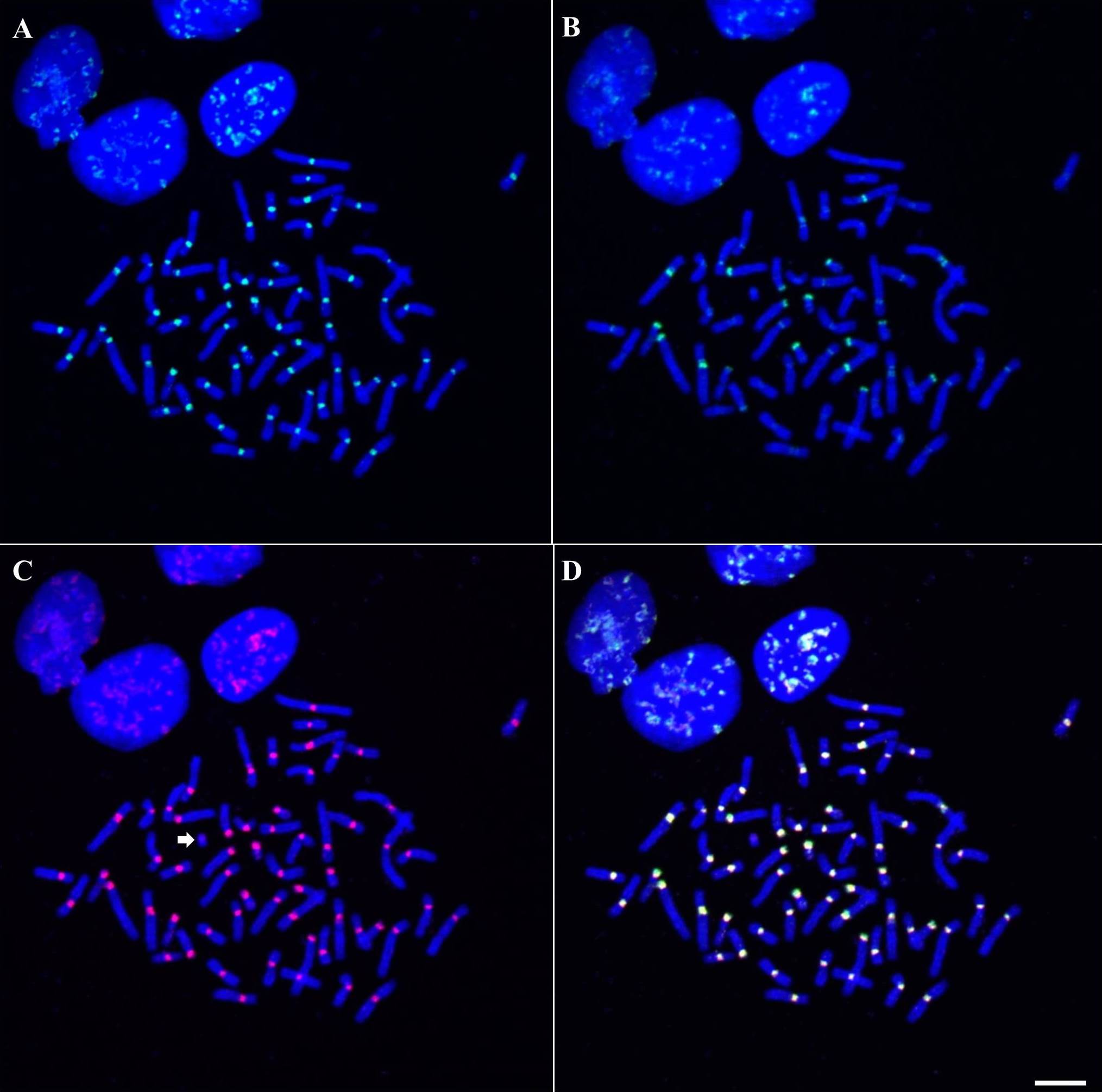


**Figure S6. Comparison of the hybridization pattern of probe Cx with those of probes targeting the C1 to C4 alpha satellite families.** Probes Cx, C1b and C2b were hybridized simultaneously to *C. solatus* metaphase chromosomes, which are colored in blue. The displayed signals correspond to (A) probe C1b (shown in green), (B) probe C2b (shown in green), (C) probe Cx (shown in red), and (D) the overlap of the three signals with the same colors. Cx displays colocalization with C1b and C2b and also labels the single chromosome they do not label (arrow), which is the single chromosome strongly labeled by probes C3a/b and C4a/b. Therefore, the Cx signal overlaps with the signals of probes targeting the C1 to C4 families without extending beyond their global signal. Scale bar = 10 µm.
