# Supplementary material for: Diversity and distribution of alpha satellite DNA in the genome of an Old World monkey: Cercopithecus solatus
Source: BMC Genomics. 2016 Nov 14;17:916. doi: 10.1186/s12864-016-3246-5 (PMC5109768; doi:10.1186/s12864-016-3246-5)
Supplement: Additional file 2: Table S1. — Filtering steps from Cercopithecus solatus raw data to alpha satellite monomer and dimer datasets. Table S2. Alpha satellite family associations in Cercopithecus solatus dimer dataset. (DOCX 40 kb) [file 12864_2016_3246_MOESM2_ESM.docx]

**Table S1: Filtering steps from *Cercopithecus solatus* raw data to alpha satellite monomer and dimer datasets.**

NOTE - The numbers of sequences fitting with the filtering criteria are given (see Methods).

**Table S2: Alpha satellite family associations in *Cercopithecus solatus* dimer dataset.**

NOTE - The numbers of dimers are displayed at the intersection between left and right monomers. (A) For dimers without the XmnI site (noX), left and right monomers are not randomly associated: C2-C2 and C3-C4 over-representations are significant (Pearson's Chi-squared test; P-value < 10^-15^). (B) For dimers with the XmnI site (X), left and right monomers are not randomly associated: C1-C1 and C2-C2 over-representations are significant (P-value < 10^-4^). The sign “-“ means non-relevant associations, i.e. when the considered families are absent in the left or right monomers.
